# White Pitaya (*Hylocereus undatus*) Juice Attenuates Insulin Resistance and Hepatic Steatosis in Diet-Induced Obese Mice

**DOI:** 10.1371/journal.pone.0149670

**Published:** 2016-02-25

**Authors:** Haizhao Song, Zihuan Zheng, Jianan Wu, Jia Lai, Qiang Chu, Xiaodong Zheng

**Affiliations:** 1 College of Biosystems Engineering and Food Science, Zhejiang University, Hangzhou, P. R. China; 2 Fuli Institute of Food Science, Zhejiang University, Hangzhou, P. R. China; East Tennessee State University, UNITED STATES

## Abstract

Insulin resistance and hepatic steatosis are the most common complications of obesity. Pitaya is an important source of phytochemicals such as polyphenols, flavonoid and vitamin C which are related to its antioxidant activity. The present study was conducted to evaluate the influence of white pitaya juice (WPJ) on obesity-related metabolic disorders (e.g. insulin resistance and hepatic steatosis) in high-fat diet-fed mice. Forty-eight male C57BL/6J mice were assigned into four groups and fed low-fat diet with free access to water or WPJ, or fed high-fat diet with free access to water or WPJ for 14 weeks. Our results showed that administration of WPJ improved high-fat diet-induced insulin resistance, hepatic steatosis and adipose hypertrophy, but it exerted no influence on body weight gain in mice. Hepatic gene expression analysis indicated that WPJ supplement not only changed the expression profile of genes involved in lipid and cholesterol metabolism (*Srebp1*, *HMGCoR*, *Cpt1b*, *HL*, *Insig1* and *Insig2*) but also significantly increased the expression levels of *FGF21*-related genes (*Klb*, *FGFR2*, *Egr1* and *cFos*). In conclusion, WPJ protected from diet-induced hepatic steatosis and insulin resistance, which was associated with the improved FGF21 resistance and lipid metabolism.

## Introduction

Type 2 diabetes and non-alcoholic fatty liver disease (NAFLD) are common consequences of a combination of metabolic disorders such as dyslipidemia, hepatic steatosis, glucose intolerance and insulin resistance [1]. Mounting evidence suggests that insulin resistance and hepatic steatosis, the most common complications of obesity, are characterized by impaired energy metabolism and attributed by complex interactions between genetic background and environmental impacts such as dietary and sedentary habits[2–6]. Moreover, insulin resistance is often accompanied by hepatic steatosis [7]. Traditional pharmacotherapies for type 2 diabetes and NAFLD are often accompanied by side effects. Recently, growing evidence has suggested that natural bioactive compounds in food exerts many beneficial effects on obesity and its metabolic consequences [8–10].

Pitaya, also known as dragon fruit, is among the most important commercial tropical fruits around the world, and three varieties have been classified as *Hylocereu polyrhizus* (red pitaya), *Hylocereus undatus* (white pitaya) and *Hylocereus megalanthus* (Yellow pitaya) [11]. Pitaya is an important source of phytochemicals such as polyphenols, flavonoid and vitamin C which are related to its antioxidant activity [12, 13]. Especially the red and white pitaya have recently drawn growing attention worldwide not only because of their economic values but also their health benefits [14]. Red pitaya consumption was reported to decrease total cholesterol (TC), triglyceride (TG) and low-density lipoprotein cholesterol (LDL-C) levels while increasing the high-density lipoprotein cholesterol (HDL-C) levels in type 2 diabetic subjects. The similar lipid-improving effect of its menthol extract was found in high-fat diet-fed rats [15, 16]. In addition, the antidiabetic effect of red pitaya has also recently been demonstrated. For instance, red pitaya significantly improved insulin resistance in rats and 600 g amount of red pitaya fruit consumption every day decreased the blood glucose level in type II diabetic subjects [15, 17]. Ramli et al. also reported that supplementation of red pitaya juice could induce a trend of glucose and uric acid normalization associated with reduced ALP and ALT but increased AST levels in high-carbohydrate and high-fat diet-fed rats [18].Though both red and white pitaya are reported to be rich, natural and cost-effective source of bioactive nutrients, few studies focused on the beneficial effects of white pitaya on diabetes and NAFLD.

The objective of the present study was to investigate the influence of white pitaya juice (WPJ) on obesity-related hepatic steatosis and insulin resistance in high-fat diet-induced obese mice and explore the underlying mechanism by which pitaya juice exerted its beneficial effects. To the best of our knowledge, this is the first study to evaluate the effect of white pitaya on type II diabetes and NAFLD.

## Materials and Methods

### Chemical reagents and juice preparation

The solvents and reagents were obtained from Aladdin (Shanghai, China). Fresh white pitayas (*Hylocereus undulatus*) were purchased from agricultural and sideline products wholesale center which is located in 183 Yisheng Road, Yuhangtang District, Hangzhou. The peels were removed and the juice was squeezed out of the fruits using a juice maker. The extracted juice was then centrifuged at 10,000 g for 40 min at 4°C. After that, the supernatant was filtered, collected and stored at -80°C. The content of bioactive compounds (e.g. total polyphenol and total flavonoids) in pitaya juice were determined using the methods described previously [19, 20].

### Animals and diets

All the protocols in this study were approved by the Committee on the Ethics of Animal Experiments of Zhejiang University (Permission Number: ZJU201550501) and the experimental procedures were performed according to the National Institutes of Health regulations for the care and use of animals in research. Moreover, all efforts were made to minimize suffering. Four-week-old C57BL/6J mice (male, n = 48) were supplied by the National Breeder Center of Rodents (Shanghai, China) and housed in a 12-hour light/dark cycle environment with free access to water and food and the temperature was kept at 23±3°C with a relative humidity of 50%±10%. After one week of acclimation, the mice were randomly assigned to the following four groups (n = 12): LFD, mice fed low-fat diet with free access to water, LFDJ, mice fed low-fat diet with free accesses to WPJ; HFD, mice fed high-fat diet with free access to water and HFDJ, mice fed high-fat diet with free access to WPJ. The ingredients and energy densities of the low-fat and high-fat diets are listed in the S1 Table. Juice and water were replaced every other day and the volumes consumed were recorded. The monitoring for body weight and food consumption started in the first week and continued through the entire experiment. Food consumption of each dietary group was determined by weighing the total amount of food given on Monday and then subtracting by the amount of food remaining on Wednesday. The average food intake of each mouse was obtained by dividing the total number of the mice in the group. The low-fat diet (LFD) contained 3.85 kcal in each gram of food, and high-fat diet (HFD) contained 4.73 kcal in each gram of food. Based on the energy density and the amount of daily consumed food, the daily calorie intake was calculated. After 14 weeks of treatment, the mice were fasted overnight and sacrificed by decapitation. Blood samples were collected for serum preparation by centrifugation at 2000g for 15 minutes. The livers, hearts, kidneys, spleens, epididymal and perirenal fat and interscapular brown fat were collected, weighted and stored at -80°C prior to use.

### Biochemical analysis

Serum concentrations of glucose, TG, TC, ALT, AST, HDL-C and LDL-C were measured using an automatic biochemistry analyzer (ACCUTE TBA-40FR, Japan) according to the manufacturer’s instructions. The commercial ELISA assay kits were used to determine the serum levels of leptin (Cat^#^: MOB00), insulin (Cat^#^: E-EL-M2614c), adiponectin (Cat^#^: MRP300), LPS (Cat^#^: E-EL-0025c), FGF21 (Cat^#^: MF2100) and NPY (Cat^#^: E-EL-M0820c).

### HOMA-IR and HOMA-IS analysis

The homeostasis model assessment (HOMA) was applied to calculate the insulin resistance (HOMA-IR) and insulin sensitive index (HOMA-IS) in accordance with the following formulas:

HOMA-IR=serum glucose(mmol/L)×serum insulin(mU)/22.5

HOMA-IS=1/[serum glucose(mmol/L)×serum insulin(mU)]

### Hepatic lipid profile analysis

Liver total lipids were extracted using the Folch method with minor modifications [21]. Briefly, the liver tissues were homogenized with chloroform/methanol mixture to a final volume 15 times the volume of the sample (0.2 g in 3 ml of solvent mixture). After dispersion, the mixture was agitated for 20 min. Then the homogenate was centrifuged to recover the liquid phase. After that, the solvent was washed with 0.2 volume (0.2 ml for 1 ml) of 0.9% NaCl solution. After vortexing for some seconds, the mixture was centrifuged at 2000 rpm/min to separate the two phases. After centrifugation and removing of the upper phase, the lower chloroform phase containing total lipids was evaporated under a nitrogen stream and the lipids were resuspended using 1% Triton X-100 in chloroform. The extracting contents of TG and TC in livers were determined using commercially available kits (Elabscience, Cat^#^: E-EL-M2603c and E-EL-M2608c) according to the manufacturer's instructions.

### Histological analysis

Specimens of liver, epididymal and perirenal white adipose and interscapular brown adipose were cut and fixed in 10% buffered formalin. For oil red O staining, the liver tissues were embedded in Optimal Cutting Temperature gel and the air-dried 6μm thick sections were dipped in formalin and washed with 0.5% oil red O solution. For H&E staining, the liver and adipose tissues were embedded in paraffin, sliced at 6μm and stained with hematoxylin and eosin (H&E). The images were captured by Olympus CX41 camera (Japan). To evaluate hepatic steatosis and the adipocyte size, the morphometric analysis was performed on 3 randomly selected sections at different depths for each mouse, and the representative sections were from six mice of each individual dietary group. The two softwares ImageJ and Photoshop were used for cell counting and the quantification of adipocyte size. Initial assessment of adipocyte size was performed under lower magnification (40×, containing> 300 cells) and confirmed under higher magnification (100×, containing> 300 cells; or 400×, containing>50 cells). The liver steatosis was scored as follows: 0, steatosis involving <5% of fatty hepatocytes; 1, steatosis involving 5–33% of fatty hepatocytes; 2, steatosis involving 34–66% of fatty hepatocytes; 3, steatosis involving >66% of fatty hepatocytes [22].

### RNA extraction and quantitative Real-Time PCR Analysis

Total RNA was extracted from the liver with Trizol (Invitrogen Technologies, USA) according to the manufacturer’s instructions. Then the total RNA concentrations were measured by NanoDrop 2000 (Thermo Scientific, Wilmington, USA). 1μg total RNA was converted to cDNA using a reverse transcription kit (Takara, Cat^#^ RR047A). 3 pooled RNA samples were from the n = 12 total samples in which 4 mice were pooled to generate one individual sample in each dietary group. The 20μl PCR reaction mixtures consisted of 1μl cDNA, paired-primers (300nM) and 10μl SYBR® Green QPCR Master Mix (Roche). The amplification program performed on Applied Biosystems 7500 system was as follows: 3 min at 50°C, 10 min at 95°C, then 31 cycles of 15 s at 95°C and then 60s at 60°C followed by melting curve for 60s at 95°C, then gradual decrease to 50°C, 20 s at 50°C, then gradually increase to 95°C, and last 20 s.

The primers are presented in the S2 Table. The relative expression of mRNA was normalized using the geometric mean of 5 housekeeping genes, including β-actin, RPS18, B2M, TBP and ARBP and the relative fold change was calculated using the 2^-△△Ct^ method.

### Statistical analysis

Values were expressed as means ± SEM. The statistical analyses were performed using SPSS 19.0 statistical software and the differences among multiple groups were assessed by one-way analysis of variance (ANOVA) followed by Duncan’s post-hoc test. p< 0.05 was considered statistically significant.

## Results

### Characterization of white pitaya juice

The bioactive compounds contained in WPJ were listed in Table 1.

**Table 1:** Chemical characterization of white pitaya juice.

Component	Amount (g / L)
Protein	8.26±0.25
Total sugar	60.42±4.16
Fiber	2.03±0.05
Total polyphenols	0.32±0.02 GAE
Total flavonoids	0.27±0.03 RE
Vitamin C	0.08±0.004

GAE, gallic acid; RE, rutin.

### Effect of WPJ on body weight gain, tissue weights, food intake, liquid intake and serum lipid profile

As depicted in Table 2, high-fat diet feeding induced a great body weight gain in mice, but no significant differences in either final body weight or the cumulative weight gain were observed between the HFDJ and HFD group. The similar results were found between LFD and LFDJ group.

**Table 2:** Body weights, cumulative weight gains, tissue weights and serum parameters in male C57BL/6J mice.

Items	LFD	LFDJ	HFD	HFDJ
Initial BW (g)	19.18±0.47	19.48±0.38	19.74±0.64	18.76±0.51
Final BW (g)	27.31±0.73^a^	26.92±0.57^a^	46.32±0.85^b^	44.69±0.87^b^
Cumulative weight gain (g)	8.14±0.91^a^	7.44±0.68^a^	26.58±0.99^b^	25.93±0.66^b^
Food intake (g/mouse/day)	3.09±0.08^a^	3.19±0.08^a^	2.54±0.09^b^	2.53±0.07^b^
Calorie intake (kcal/mouse/day)	11.9±0.31	12.28±0.29	12±0.44	11.98±0.33
Liquid intake (ml/mouse/day)	2.99±0.08^a^	3.3±0.07^b^	2.61±0.06^c^	2.98±0.07^a^
**Tissue weight (g)**				
Heart	0.15±0.01	0.16±0.01	0.16±0.01	0.17±0.02
Liver	1.04±0.06^a^	0.97±0.05^a^	1.44±0.06^b^	1.16±0.05^a^
Spleen	0.06±0.004^a^	0.05±0.003^a^	0.08±0.004^b^	0.07±0.003^c^
Kidney	0.33±0.01^a^	0.33±0.01^a^	0.44±0.02^b^	0.38±0.02^a^
Perirenal WAT	0.18±0.03^a^	0.2±0.02^a^	1.9±0.11^b^	1.67±0.09^c^
Epididymal WAT	0.41±0.07^a^	0.39±0.08^a^	2.67±0.15^b^	2.08±0.1^c^
Interscapular BAT	0.15±0.01	0.15±0.02	0.22±0.04	0.18±0.02
**Tissue index (% BW)**				
Heart	0.57±0.03^a^	0.6±0.04^a^	0.34±0.01^b^	0.38±0.02^b^
Liver	3.8±0.21^a^	3.64±0.24^ab^	3.13±0.16^b^	2.6±0.14^c^
Spleen	0.21±0.01^a^	0.2±0.01^ab^	0.18±0.01^bc^	0.16±0.01^c^
Kidney	1.23±0.06^a^	1.22±0.06^a^	0.96±0.05^b^	0.84±0.04^b^
Perirenal WAT	0.65±0.1^a^	0.72±0.08^a^	4.1±0.21^b^	3.75±0.23^b^
Epididymal WAT	1.46±0.23^a^	1.41±0.25^a^	5.75±0.29^b^	4.46±0.23^c^
Interscapular BAT	0.56±0.04	0.56±0.08	0.48±0.09	0.41±0.05
**Serum parameters**							
TG (mmol/L)	0.89±0.08^a^	0.68±0.06^b^	1.44±0.08^c^	1±0.07^a^
TC (mmol/L)	3.09±0.29^a^	3.35±0.23^a^	5.77±0.31^b^	3.79±0.23^a^
HDL-C (mmol/L)	2.61±0.18^a^	2.78±0.2^a^	5.01±0.3^b^	4.58±0.13^b^
LDL-C (mmol/L)	0.41±0.02^a^	0.37±0.03^a^	0.76±0.04^b^	0.28±0.03^c^
AST (U/L)	139.8±8.94^a^	137.7±10.19^a^	185.4±8.19^b^	156.7±8.17^a^
ALT (U/L)	39±2.93^a^	27.3±2.18^a^	109.5±5.08^b^	57.8±7.71^c^

LFD, mice fed low-fat diet; LFDJ, mice fed low-fat diet with supplementation of WPJ; HFD, mice fed high-fat diet; HFDJ, mice fed high-fat diet with supplementation of WPJ. BW, body weight; WAT, white adipose; BAT, brown adipose; TG, triglyceride; TC, total cholesterol; ALT, Alanine aminotransferase; AST, aspartate aminotransferase; HDL-C, high-density lipoprotein cholesterol; LDL-C, low-density lipoprotein cholesterol. Values are presented as means ± SEM (n = 10). Data not sharing a common superscript letter differ significantly among groups (p<0.05, ANOVA).

In addition, mice in LFD and LFDJ group consumed more food than those in HFD and HFDJ group, and mice in LFDJ and HFDJ group intake more liquid than those in LFD and HFD group. But there were no differences in the calorie intake among all the dietary groups. In addition, according to the FDA ratio of conversion, the human-equivalent dose for pitaya juice based on body surface area is approximately 440ml for a 60Kg human. High-fat diet-induced a decrease in the relative weight of heart, liver and kidney but a significant increase in the relative weight of perirenal and epididymal white adipose and no influence on spleen and interscapular brown adipose compared to mice fed low-fat diet (Table 2). No difference in the organ weights existed between the LFD and LFDJ group. The same results were also found between the HFD and HFDJ group, except a decrease in the weight of epididymal white adipose in HFDJ group. Moreover, the serum levels of TG, TC, HDL-C, LDL-C, ALT and AST were significantly increased in mice fed high-fat diet compared to those fed low-fat diet, while administration of WPJ significantly reduced the levels of TG, TC, LDL-C, ALT and AST but not HDL-C. In addition, WPJ also tended to decrease TG and ALT in mice fed low-fat diet (Table 2).

### Effect of WPJ on serum cytokines

**Fig 1:**
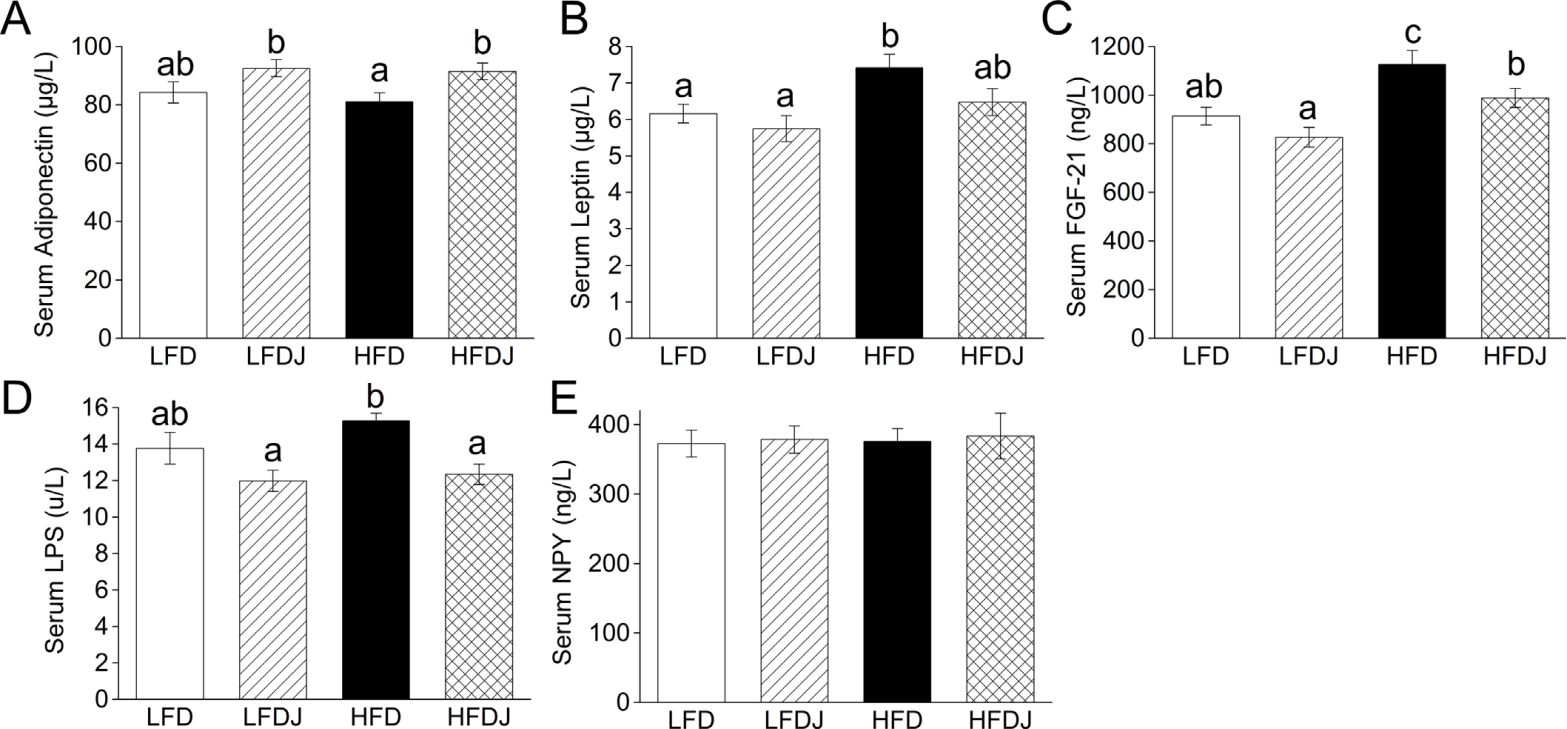
Effect of white pitaya juice (WPJ) on serum cytokines. The fasting serum levels of adiponectin (A) and leptin (B). (C) Circulating levels of *FGF21*. The fasting serum levels of LPS (D) and NPY (E). *FGF21*, fibroblast growth factor 21; NPY, neuropeptide Y; LFD, mice fed low-fat diet; LFDJ, mice fed low-fat diet with supplementation of WPJ; HFD, mice fed high-fat diet; HFDJ, mice fed high-fat diet with supplementation of WPJ. n = 10 (A, B, C and D). Values are presented as means ± SEM. Data not sharing a common superscript differ significantly among groups (p<0.05, ANOVA).

WPJ supplementation significantly reduced high-fat diet feeding-induced increase of serum levels of *FGF21* and LPS and also tend to decrease the leptin level, although not significant (Fig 1B, 1C and 1D). Though there is not a significant increase in the serum level of adiponectin in LFDJ group compared to that in LFD group, the level of serum adiponectin in the HFDJ group was significantly higher than that in the HFD group (Fig 1A). In addition, neither high-fat diet feeding nor WPJ administration influenced the serum levels of and NPY (Fig 1E).

### WPJ alleviates obesity-related hepatic steatosis and adipose tissue hypertrophy

**Fig 2:**
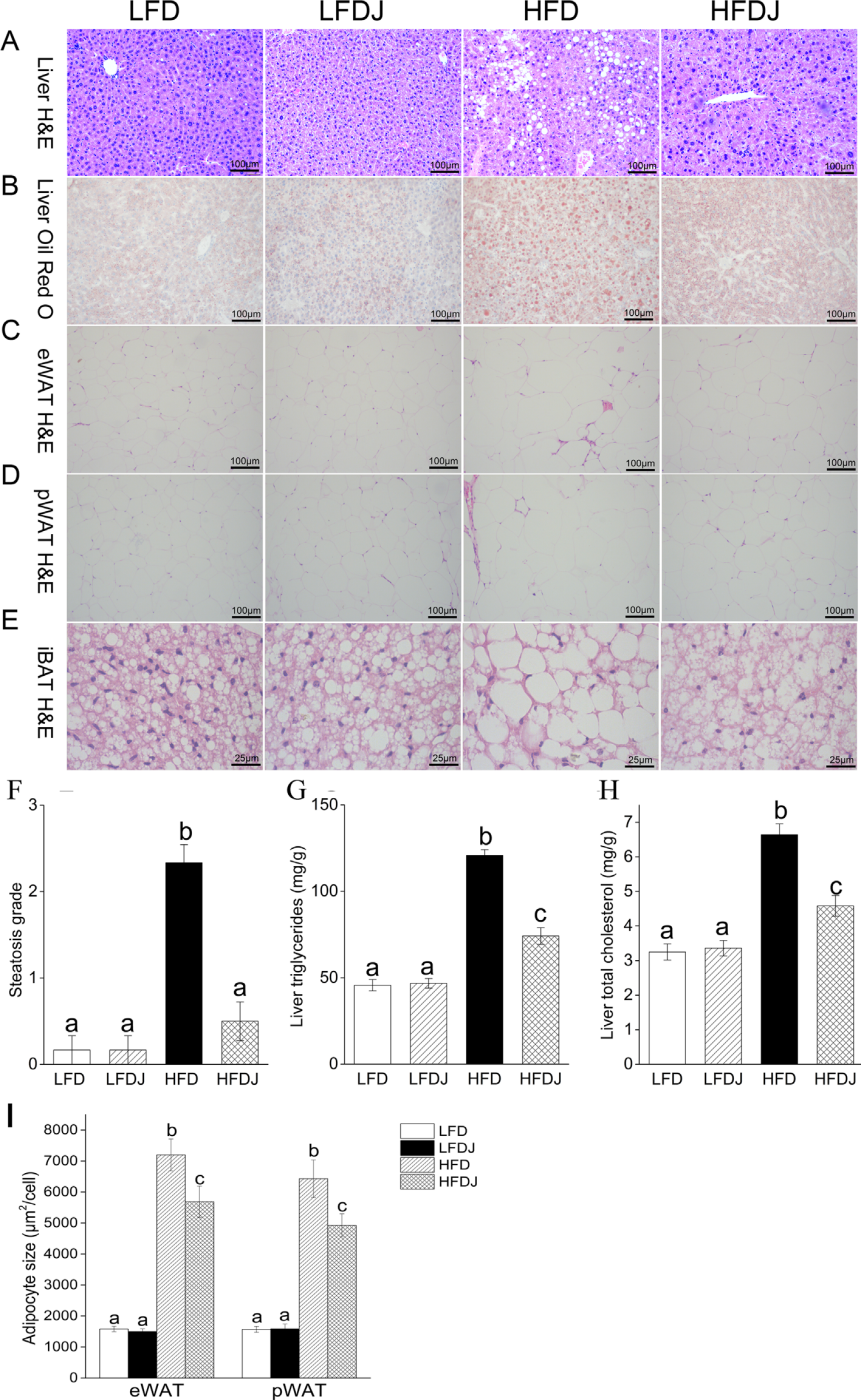
White pitaya juice (WPJ) supplementation attenuates high-fat diet-induced hepatic steatosis and adipocyte hypertrophy. H&E staining (A) and oil red O staining (B) of liver tissues. H&E staining of epididymal white adipose (C), perirenal white adipose (D) and interscapular brown adipose (E). (F) Hepatic steatosis grade. (G) Hepatic triglycerides levels. (H) Hepatic cholesterol levels. eWAT, epididymal white adipose; pWAT, perirenal white adipose; iBAT, interscapular brown adipose; LFD, mice fed low-fat diet; LFDJ, mice fed low-fat diet with supplementation of WPJ; HFD, mice fed high-fat diet; HFDJ, mice fed high-fat diet with supplementation of WPJ. n = 6 (F), n = 10 (G and H). Values are presented as means ± SEM. Data not sharing a common superscript differ significantly among groups (p<0.05, ANOVA).

H&E and Oil red O staining of livers revealed an obvious hepatic lipid droplets accumulation in mice fed high-fat diet and the mice developed high degree of hepatic steatosis with swelling of hepatocytes and severe cytoplasmic vacuoles, while the supplementation of WPJ significantly alleviated lipid accumulation in livers and greatly improved liver steatosis (Fig 2A and 2B). The liver steatosis grades were significantly lower in the HFDJ group than that in the HFD group (Fig 2F). Consistent with these results, WPJ supplementation also significantly decreased high-fat diet-induced elevation in the hepatic TG and TC levels (Fig 2G and 2H).

Histological analysis also showed that WPJ reduced the cell size of white adipocyte tissue (WAT) (Fig 2C, 2D and 2I) and brown adipocyte tissue (BAT) (Fig 2E). No significant abnormalities were observed in the LFD and LFDJ group.

### WPJ improves diet-induced insulin resistance

**Fig 3:**
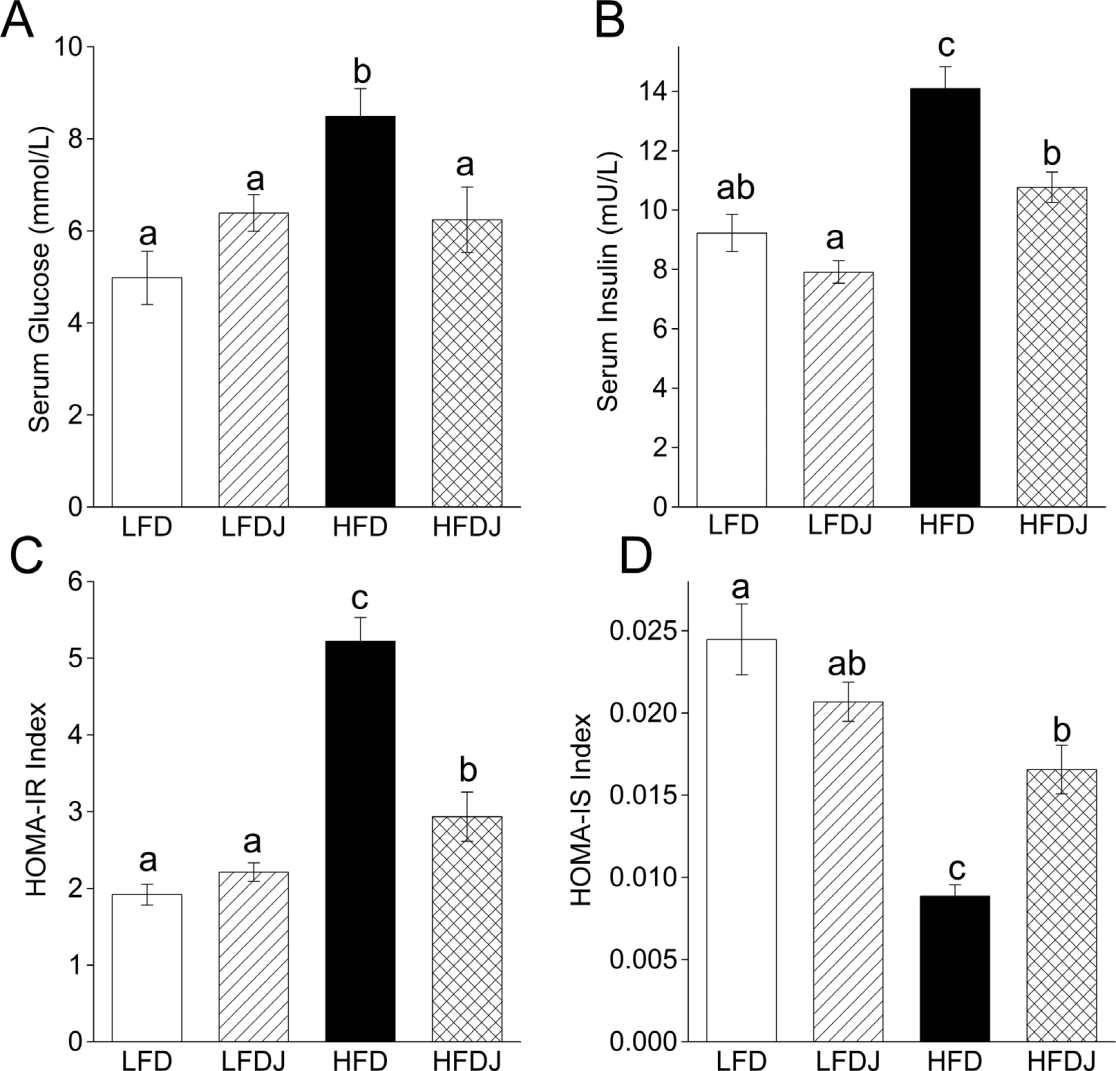
White pitaya juice (WPJ) improves high-fat diet-induced hyperglycemia and insulin resistance. (A) The fasting serum levels of glucose. (B) The fasting serum levels of insulin. (C) HOMA-IR index. (D) HOMA-IS index. LFD, mice fed low-fat diet; LFDJ, mice fed low-fat diet with supplementation of WPJ; HFD, mice fed high-fat diet; HFDJ, mice fed high-fat diet with supplementation of WPJ. n = 10 (A), n = 10 (B, C and D). Values are presented as means ± SEM. Data not sharing a common superscript differ significantly among groups (p<0.05, ANOVA).

High-fat diet induced an increase in the fasting serum levels of glucose and insulin, accompanied by an increase of HOMA-IR and decrease of HOMA-IS index, while the mice in HFDJ group revealed a significant decrease in the fasting serum glucose and insulin levels and HOMA-IR index but a distinct increase of HOMA-IS index compared to mice in HFD group (Fig 3), suggesting that WPJ significantly improved high-fat diet-induced hyperglycemia and insulin resistance in mice. No significant differences were observed between the LFD and LFDJ group.

### WPJ alters the hepatic gene expression profiles

**Fig 4:**
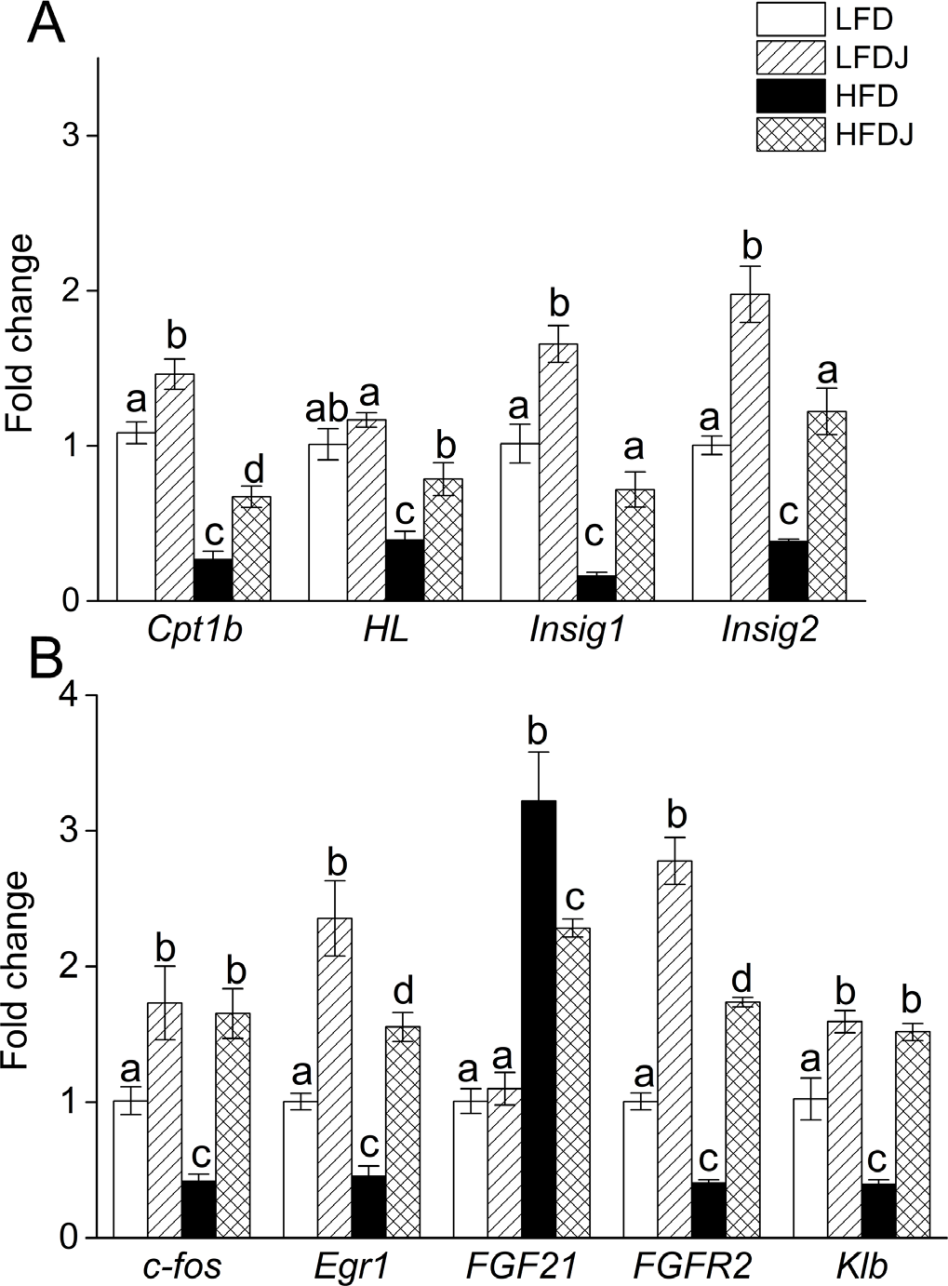
White pitaya juice (WPJ) changes the hepatic gene expression profile. (A) The relative expression levels of genes involved in lipid metabolism and in cholesterol biosynthesis in livers. (B) The relative expression levels of fibroblast growth factor 21-related genes in livers. *Cpt1b*, carnitine palmitoyltransferase 1b; *HL*, hepatic lipase; *Insig1*, insulin-induced gene 1; *Insig2*, insulin-induced gene 2; *FGF21*, fibroblast growth factor 21; *Klb*, β-klotho; *FGFR2*, fibroblast growth factor receptor 2; LFD, mice fed low-fat diet; LFDJ, mice fed low-fat diet with supplementation of WPJ; HFD, mice fed high-fat diet; HFDJ, mice fed high-fat diet with supplementation of WPJ. Values are presented as means ±SEM. Data not sharing a common superscript differ significantly among groups (p<0.05, ANOVA).

For genes involved in fatty acid metabolism, WPJ supplementation significantly increased the expression levels of *Cpt1b* (Carnitine palmitoyltransferase 1b) and *HL* (Hepatic lipase) (Fig 4A). Similarly, the expression levels of genes involved in cholesterol biosynthesis, including insulin-induced gene 1, 2 (*Insig1* and *Insig2*) were reduced by high-fat diet feeding but significantly increased by WPJ treatment (Fig 4B). In addition, WPJ treatment suppressed the expression of *HMGCoR* (Hydroxy-3-Methylglutaryl-Coenzyme A Reductase) and *Srebp-1*(Sterol regulatory element-binding transcription factor 1), but no significant changes in the expression of *Pparα* (Peroxisome proliferator-activated receptor alpha), *Pparγ* (Peroxisome proliferator-activated receptor gamma) and *LDFR* (The Low-Density Lipoprotein Receptor) were observed (S1 Fig). Moreover, high-fat diet feeding increased the expression level of *FGF21* and it suppressed the expression of its receptors, *Klb* (*β-Klotho*) and *FGFR2* (fibroblast growth factor receptor 2) as well as its target genes, including *Egr1* and *cFos*. However, WPJ treatment decreased the expression level of fibroblast growth factor 21 (*FGF21*), but it significantly increased the expression levels of *Klb*, *FGFR2*, *Egr1* and *cFos* (Fig 4C).

## Discussion

Increasing evidence indicates that the consumption of vegetables and fruits is inversely related to many metabolic disorders, thus decreasing the risk of type II diabetes, NAFLD and obesity, and the beneficial health effects of these plant foods are due to their high content of polyphenols, flavonoids, vitamins, dietary fibers and minerals [23–26]. Pitaya, which are mainly cultivated in East and Southeast Asia, Central and South America and northern Australia, is reported to be a natural source of bioactive nutrients, but few studies focused on its beneficial effects on metabolic syndromes. The present study was conducted to investigate the effects of WPJ on type-2 diabetes and NAFLD in high-fat diet-induced obese mice. Our results clearly demonstrated that WPJ supplementation significantly attenuated diet-induced hepatic steatosis as revealed by reduced hepatic lipid accumulation, and the beneficial effect was accompanied by the improved insulin resistance and insulin sensitivity, as revealed by lower fasting serum glucose and insulin levels and a lower HOMA-IR index but higher HOMA-IS index. Moreover, WPJ administration did not affect the body weight gain and the calorie intake of mice, but it decreased the relative weight of epididymal white adipose and attenuated high-fat diet-induced adipose hypertrophy, as revealed by the downsized white and brown adipocyte.

Human and animal studies have proved the lipid-improving and antidiabetic effect of red pitaya [15–17]. Considering the difference of constituents in white and red pitaya, especially the absence of betacyanins in white pitaya, the beneficial effect of white pitaya needs to be clarified. And consistent with the studies focused on red pitaya, our results indicated that WPJ supplementation significantly decreased the serum levels of TG, TC and LDL-C in high-fat diet fed mice, suggesting the lipid -reducing effect of WPJ. In addition, we observed that WPJ supplementation did not induce a weight loss in high-fat diet-fed mice, which was consistent with the previous results, and we postulated that this condition might be due to the high content of sugar and fat in WPJ that counteracted the weight-loss effect. Despite of no influence on body weight gain, WPJ administration significantly reduced the size of white and brown adipocytes, as revealed by histological analysis of adipose, suggesting that WPJ could attenuate high-fat diet-induced adipose tissue hypertrophy, which has not been reported previously. In addition, there was no difference in the daily calorie intake among all the dietary groups. Consistent with these results, the serum levels of NPY, which could regulate food intake and storage of energy [27], were not affected by HFD feeding or WPJ intervention, suggesting that WPJ exerted its beneficial effects on lipid metabolism without affecting the energy intake of mice.

Leptin plays an important role in regulating energy homeostasis and lipid metabolism, and most of obese animals exhibit a leptin-resistant status [28]. Consistently, the leptin level of HFD-fed mice has been elevated and WPJ treatment induced a clear decrease, although not significant, in the serum level of leptin, implying that WPJ administration might improve HFD-induced leptin-resistance, directly or indirectly. Furthermore, we also identified elevated serum levels of adiponectin in the WPJ-treated HFD mice. Adiponectin has been proved to regulate the metabolism of lipids and glucose [29]. These findings implied that the beneficial effects of WPJ on these metabolic syndromes might be associated with the improved lipid metabolism.

Hepatic lipid accumulation is a biomarker of liver steatosis, which in combination with oxidative stress and inflammation can lead to NAFLD [30]. Few studies have been conducted to assess the effect of pitayas intake on the obesity-related NAFLD. In this study, H&E staining of livers revealed that WPJ supplementation reduced the hepatic lipid accumulation, alleviated the swelling of hepatocytes and prevented the formation of liver steatosis in high-fat diet-fed mice. Consistently, WPJ administration also decreased the hepatic TG and TC levels as well as the serum ALT and AST levels. To the best of our knowledge, this was the first study reported that pitaya possessed anti-hepatosetatosis effect.

Hepatic gene expression analysis indicated that WPJ treatment changed the expression profile of the genes (*Cpt1b*, *LPL* and *Srepb1*) involved in fatty acid metabolism and the genes (*Insig1*, *Insig2* and *HMGCoR*) involved in cholesterol biosynthesis. These genes have been fully elucidated to play crucial roles in the modulation of cholesterol biosynthesis, fatty acid biosynthesis or beta-oxidation [31–35]. Lipopolysaccharides (LPS), also known as lipoglycans and endotoxins. High-fat diet has been proved to induce elevated levels of serum LPS, a condition referred to as endotoxemia. The elevated levels of serum LPS triggers several inflammatory markers which are a precursor to the progression towards metabolic syndrome, resulting in significant inflammation, body weight gain, insulin resistance, and ultimately type-2 diabetes [36, 37]. Accordingly, the bioactivity of white pitaya juice to blunt circulating LPS levels may be accounted for the reduced lipid accumulation in livers and the protection from NAFLD and insulin resistance in high-fat diet-fed mice. Regarding these considerations, we decided to examine the serum levels of LPS. Consistent with the previous studies, 14 weeks of HFD feeding induced a significant increase in circulating LPS level, which was fully prevented by WPJ supplementation. This finding was associated with the reduced serum and hepatic TG levels by WPJ treatment.

Obesity and hepatic steatosis are usually accompanied by insulin resistance, therefore increasing the risk of type II diabetes [7, 38], and insulin resistance in obese diabetic II subjects is worse than that in non-obese diabetic subjects [39]. The blood glucose-controlling and hyperinsulinemia-improving effects of red pitaya have been reported respectively in diabetic patients and rats [15, 17]. However, Ramli et al. recently reported that red pitaya juice exert no influence on blood glucose levels in high-carbohydrate and high-fat diet-fed rats [18]. So the anti-diabetic effect of red pitaya seems controversial. In this study, we found that WPJ administration not only significantly decreased the serum levels of glucose and insulin but also decreased the HOMA-IR index, suggesting that WPJ improved high-fat diet-induced insulin resistance in mice. Moreover, the increased HOMA-IS index in WPJ-treated mice also implied the insulin-sensitizing effect of WPJ. These results indicated a strong anti-diabetic activity of white pitaya.

FGF21, as a novel member of the FGFs family, is mainly expressed in liver [40]. A growing body of literatures show that exogenous FGF21 administration improve glucose and lipid homeostasis, insulin sensitivity and hepatic steatosis [41, 42]. However, mounting studies also support that plasma FGF21 levels are markedly increased in obesity, insulin-resistant, hypertriglyceridemia, hepatosteatosis and liver injury states [43, 44], suggesting that FGF21 did not exert its beneficial effects on lipid and glucose homeostasis. The failure of endogenous FGF21 to improve these metabolic syndromes implied a FGF21-resistant state due to impaired FGF21 action. Consistent with the previous findings, our results showed that high-fat diet feeding induced an increase of serum level of FGF21, which implied that obese state could result in a compensatory overproduction of FGF21. Numerous studies have proven that FGF21 activity depends on binding to its cofactor, β-Klotho, (Klb) and its receptors (FGFRs) [45]. Our results showed that high-fat diet feeding increased the hepatic expression level of FGF21, but it significantly suppressed the expression of FGFR2 and Klb. High-fat diet also significantly resulted in the impaired induction of Egr1 and cFos which are the target genes of FGF21. Though WPJ treatment decreased the expression FGF21, it significantly increased the expression levels of Klb, FGFR2, Egr1 and cFos. These data suggested that WPJ could improve the expression profile of FGF21 receptor and cofactor, thus attenuating FGF21 resistance. So one potential action site for WPJ to exert its anti-diabetic and anti-hepatosteatosis effect might be restoring the function of FGF21.

In summary, our results demonstrated that WPJ administration improved diet-induced insulin resistance and hepatic steatosis in mice. The beneficial effects of WPJ on these metabolic syndromes were associated with the improved FGF21 resistance and lipid metabolism. These findings suggest a dietary choice in the management of type II diabetes and NAFLD.

## Supporting Information

S1 Fig The influence of white pitaya juice (WPJ) on the expression profiles of *Pparα*, *Pparγ*, *LDLR*, *HMGCoR* and *Srebp-1*. *HMGCoR*, hydroxy-3-Methylglutaryl-Coenzyme A Reductase); *Srebp-1*, sterol regulatory element-binding transcription factor 1; *Pparα*, peroxisome proliferator-activated receptor alpha; *Pparγ*, peroxisome proliferator-activated receptor gamma; *LDFR*, low-Density Lipoprotein Receptor; LFD, mice fed low-fat diet; LFDJ, mice fed low-fat diet with supplementation of WPJ; HFD, mice fed high-fat diet; HFDJ, mice fed high-fat diet with supplementation of WPJ. Values are presented as means ±SEM. Data not sharing a common superscript differ significantly among groups (p<0.05, ANOVA).
(TIF)

S1 File Raw data. (XLSX)

S2 File Raw data. (ZIP)

S1 Table Ingredients of purified diets. (DOCX)

S2 Table Primers used for real-time quantitative PCR. (DOCX)
