# Supplementary figures and images for: White Pitaya (Hylocereus undatus) Juice Attenuates Insulin Resistance and Hepatic Steatosis in Diet-Induced Obese Mice
Source: PLoS One. 2016 Feb 25;11(2):e0149670. doi: 10.1371/journal.pone.0149670 (PMC4767368; doi:10.1371/journal.pone.0149670)

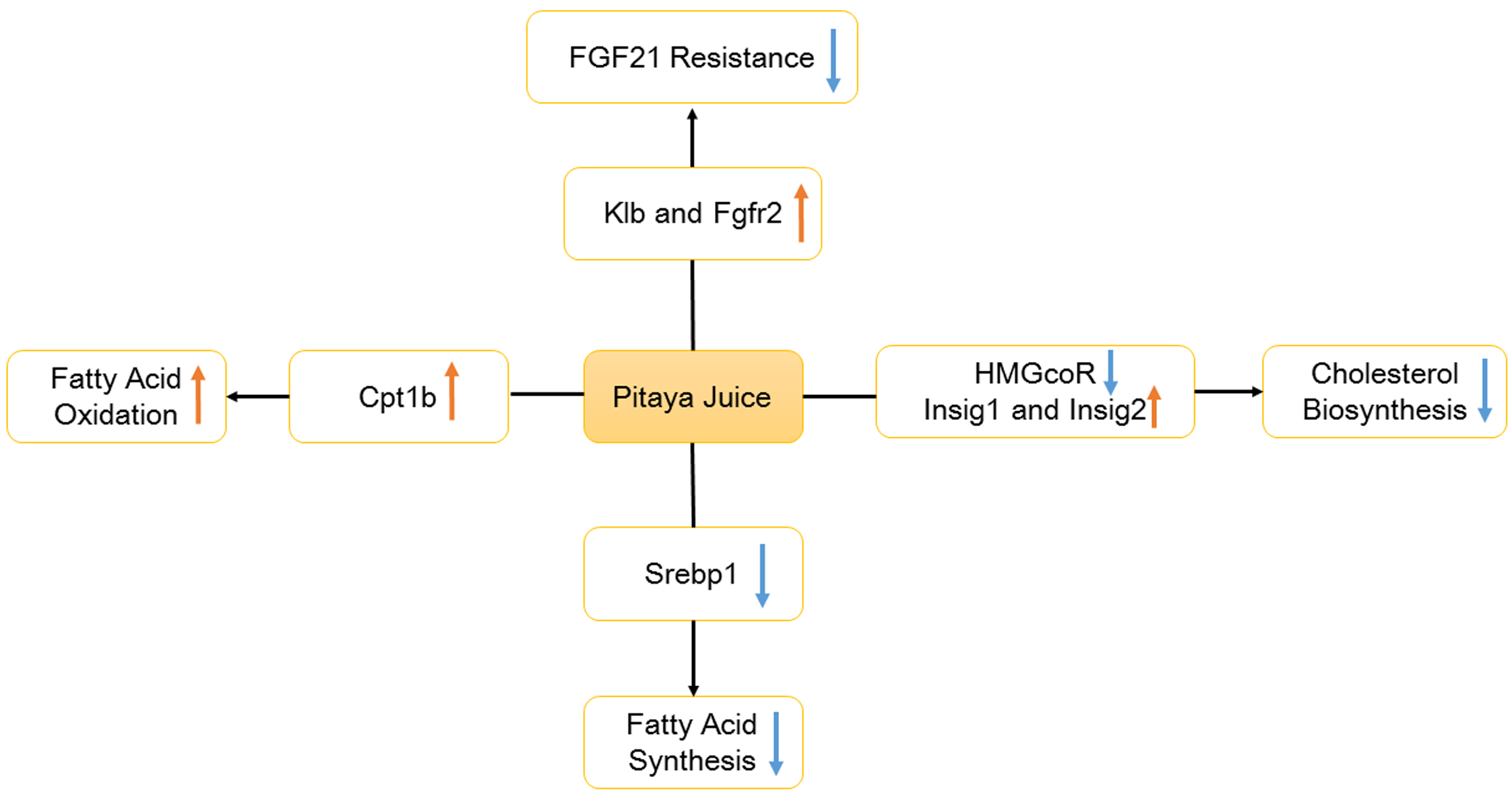

Supplement: S2 File — (ZIP) [file pone.0149670.s003.zip › S2 File/A working model.tif]

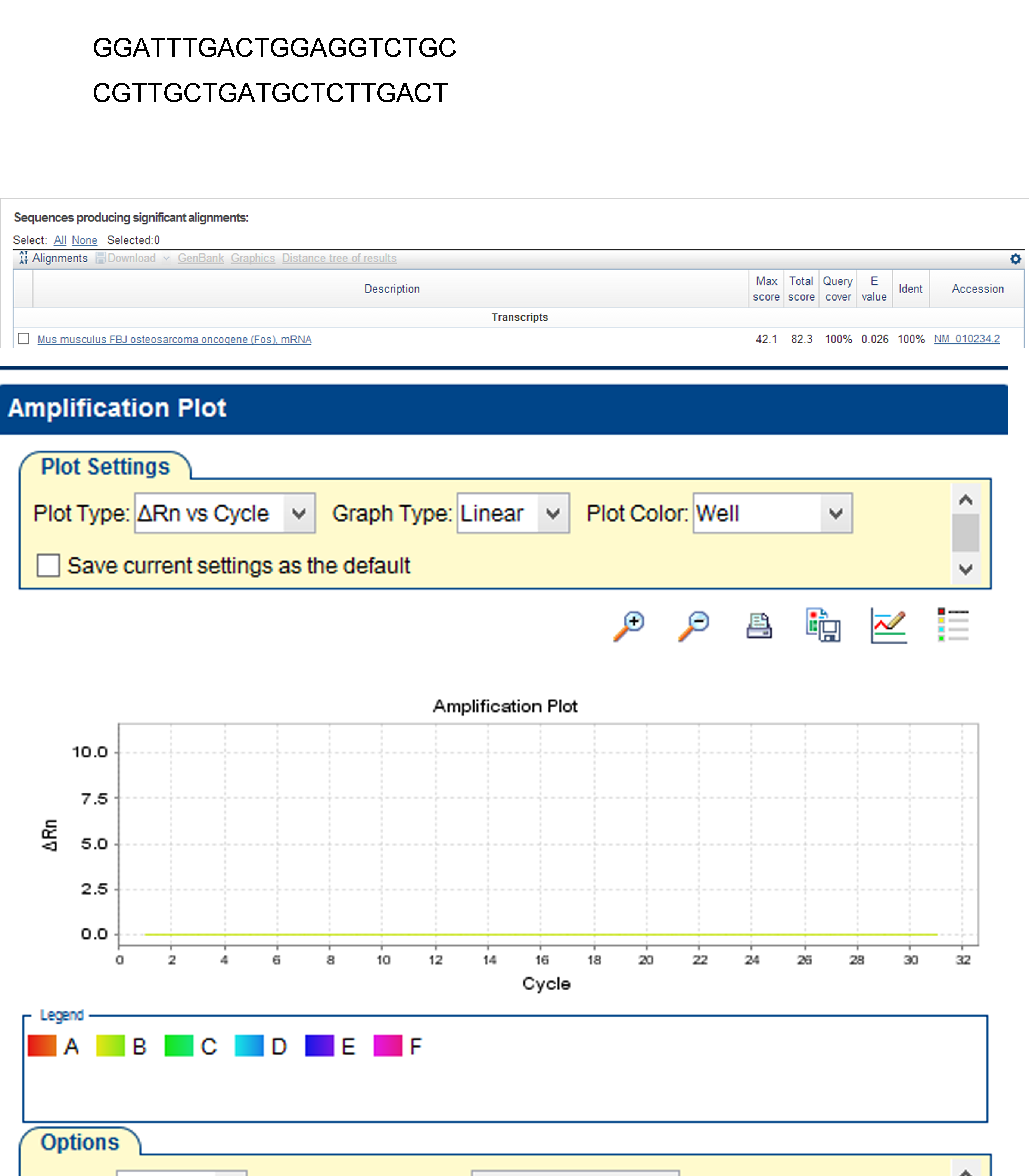

Supplement: S2 File — (ZIP) [file pone.0149670.s003.zip › S2 File/c-fos.tif]

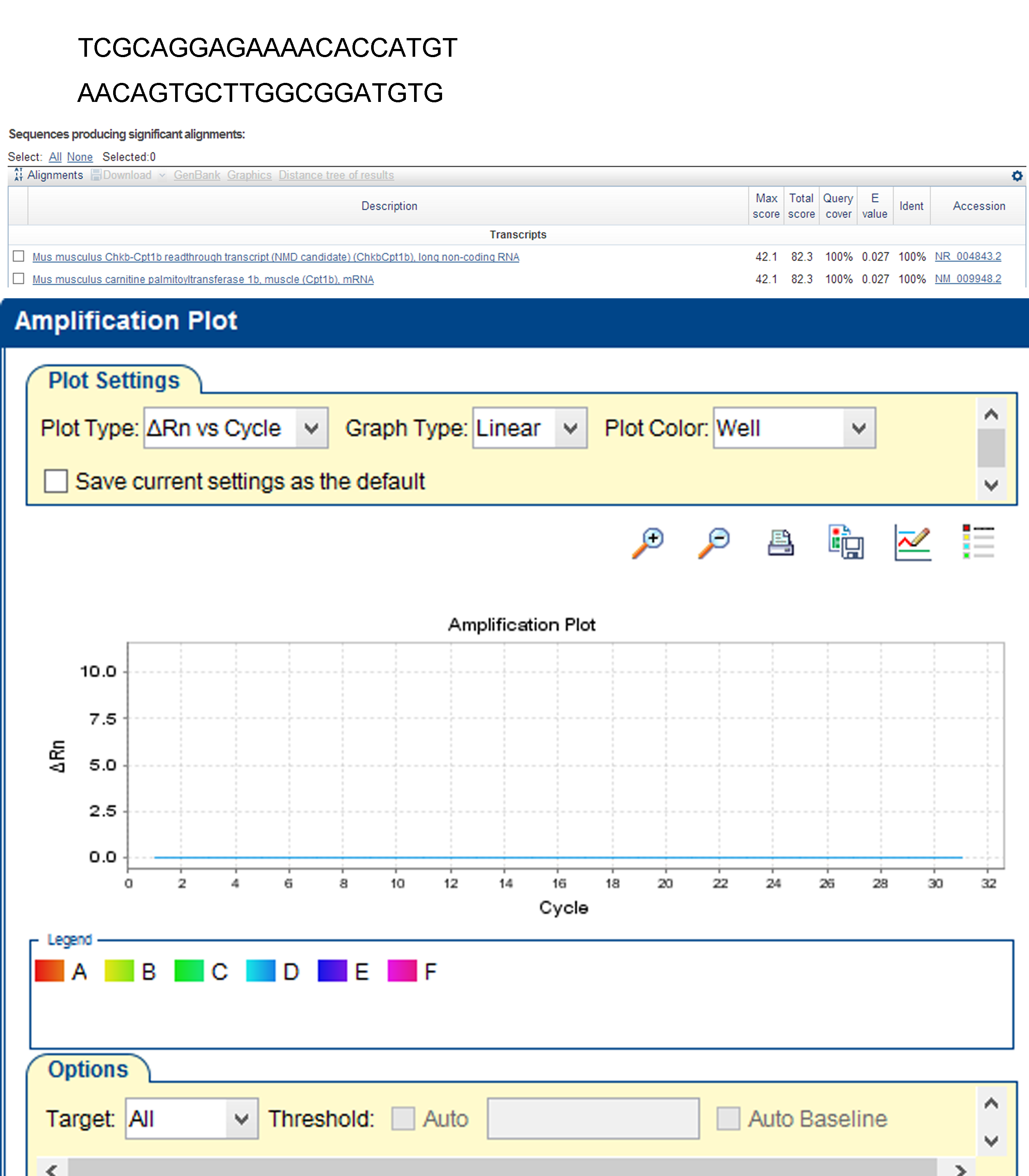

Supplement: S2 File — (ZIP) [file pone.0149670.s003.zip › S2 File/Cpt1b.tif]

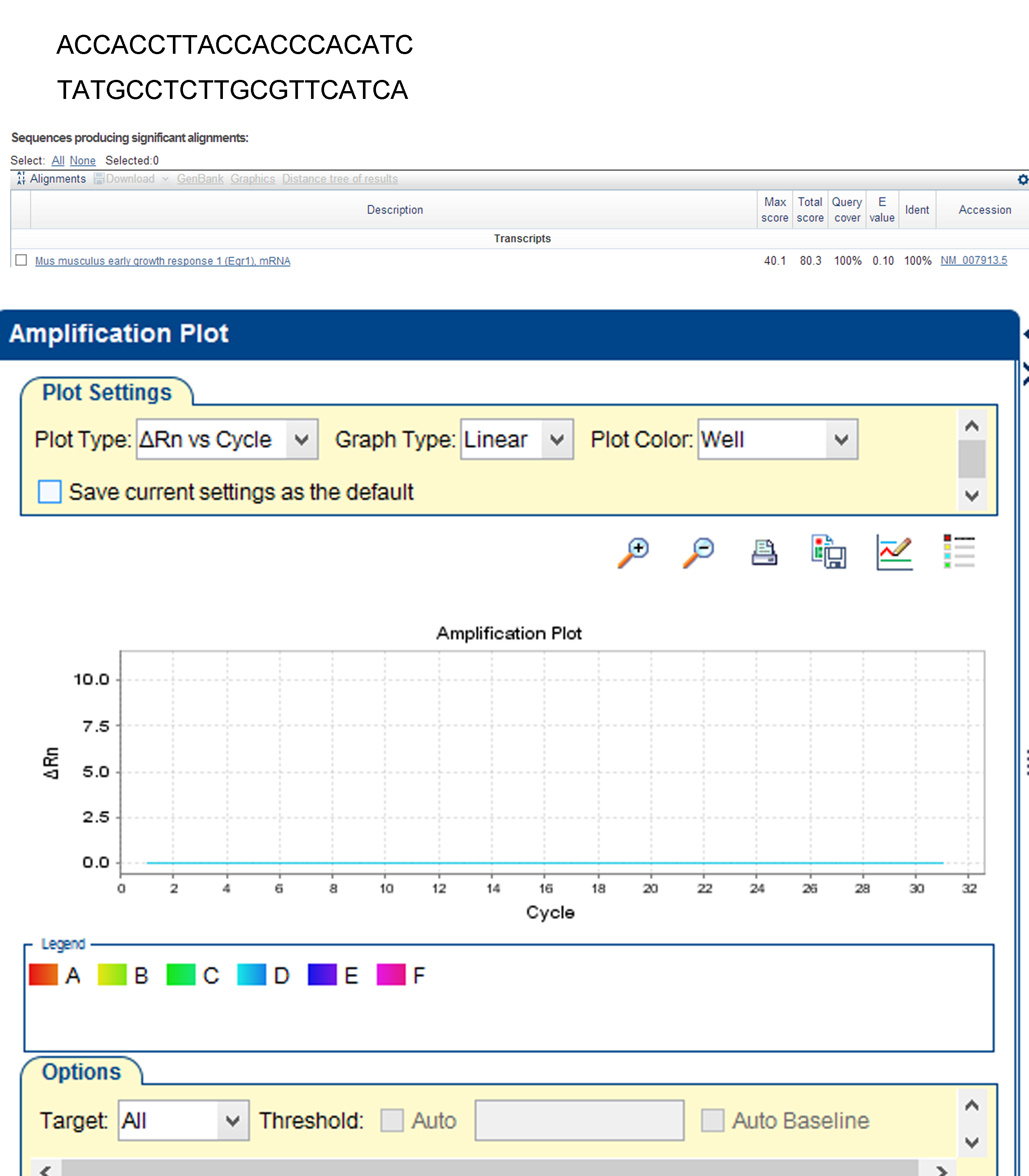

Supplement: S2 File — (ZIP) [file pone.0149670.s003.zip › S2 File/Egr1.tif]

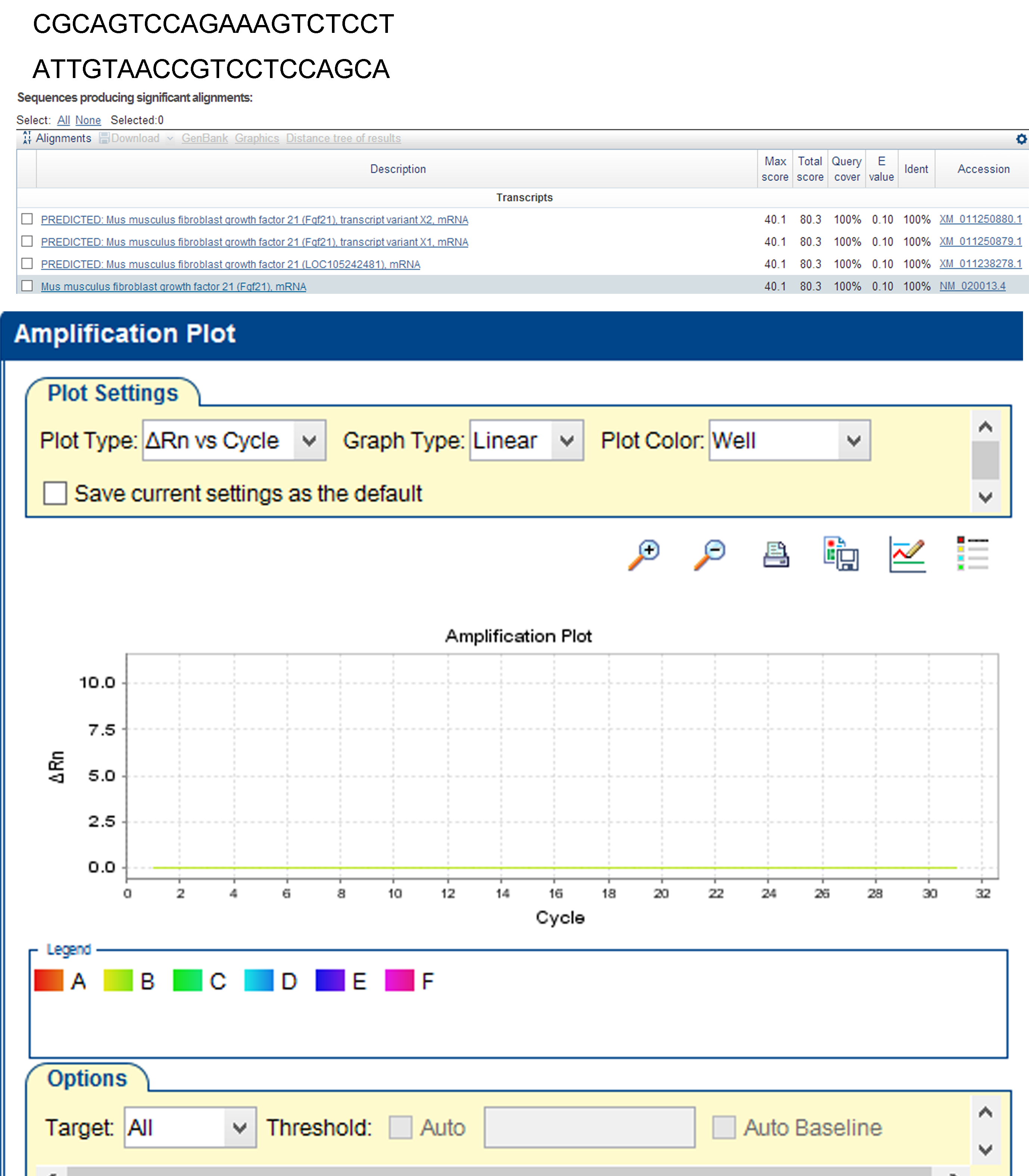

Supplement: S2 File — (ZIP) [file pone.0149670.s003.zip › S2 File/FGF21.tif]

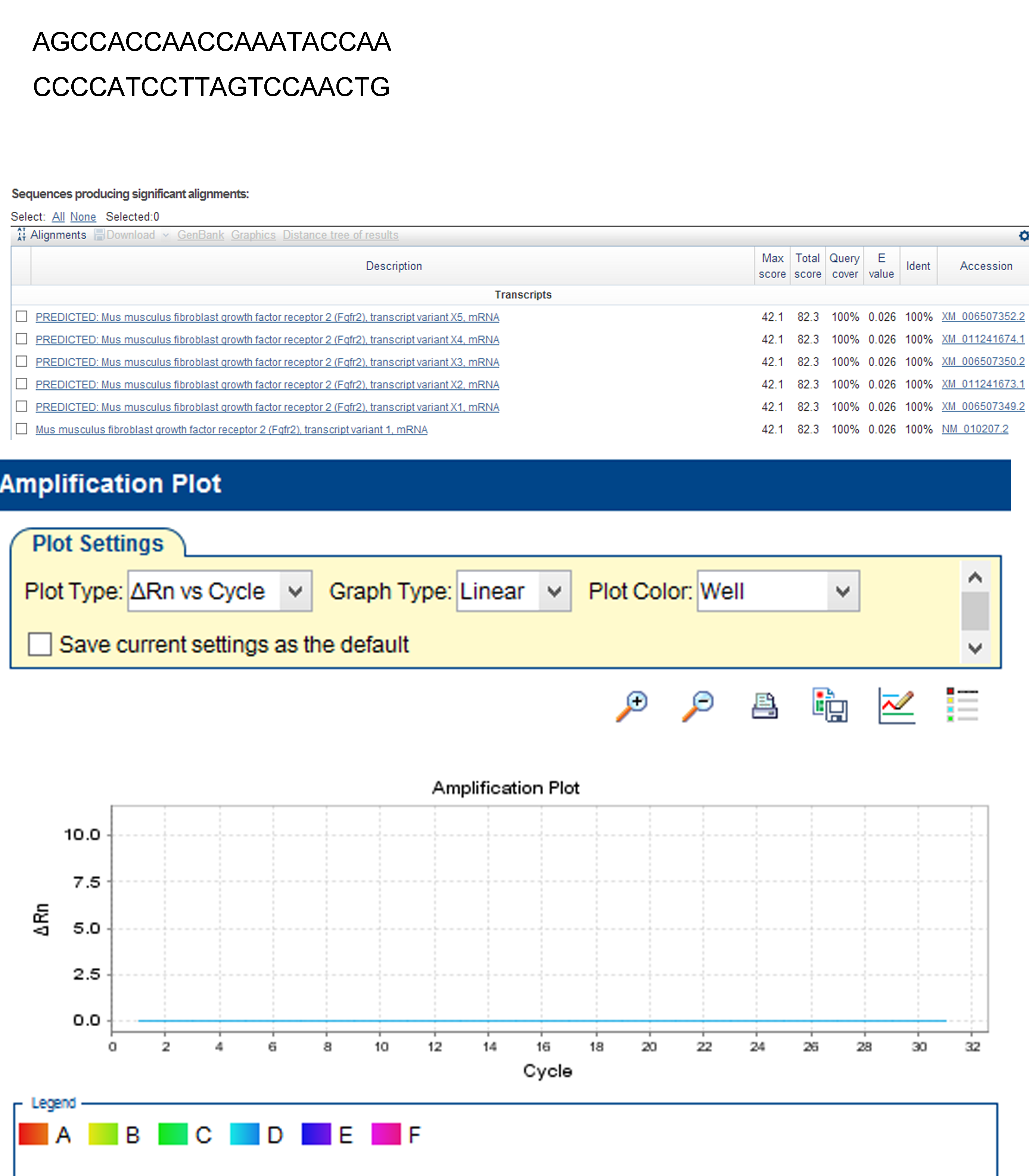

Supplement: S2 File — (ZIP) [file pone.0149670.s003.zip › S2 File/FGFR2.tif]

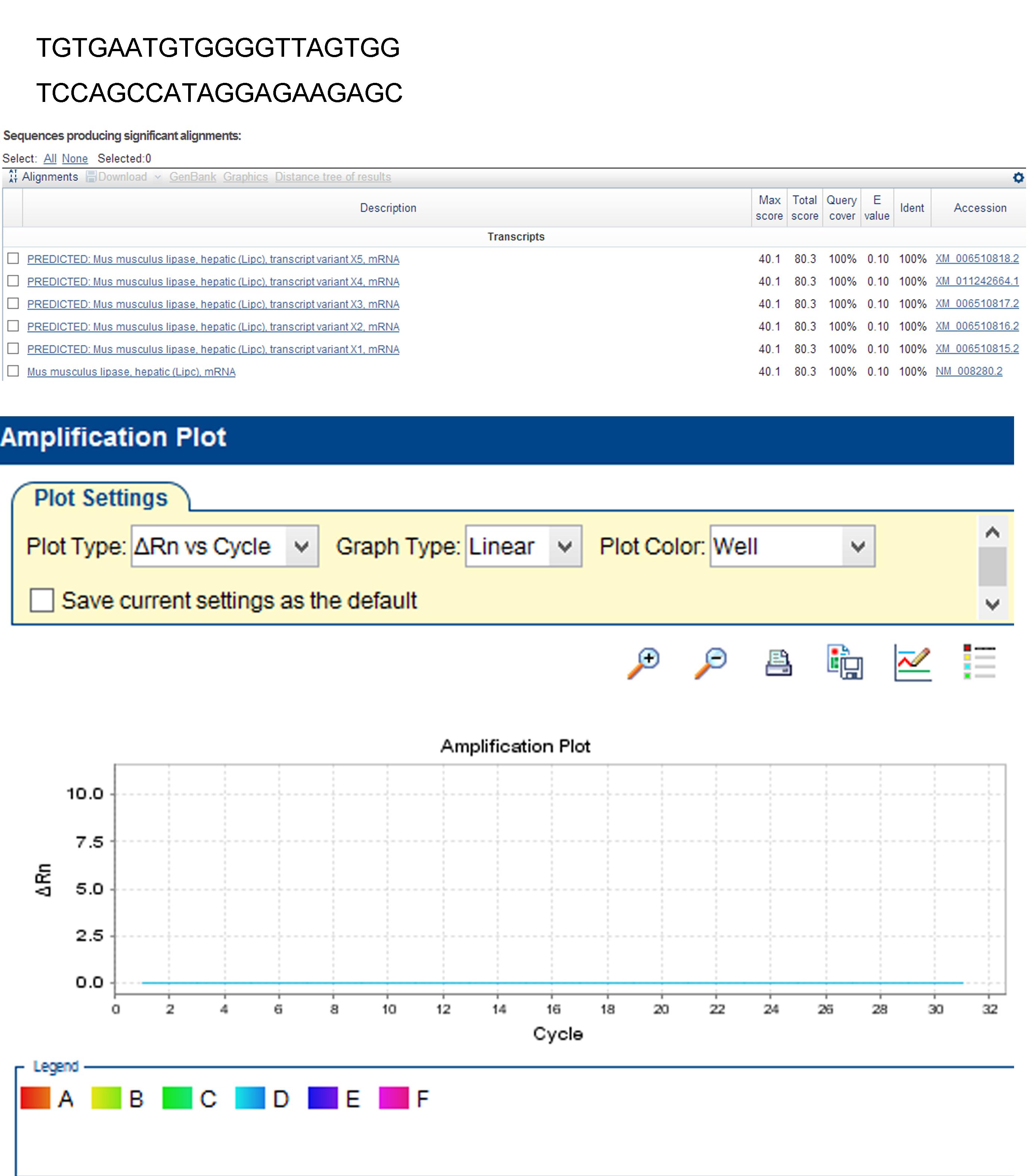

Supplement: S2 File — (ZIP) [file pone.0149670.s003.zip › S2 File/HL.tif]

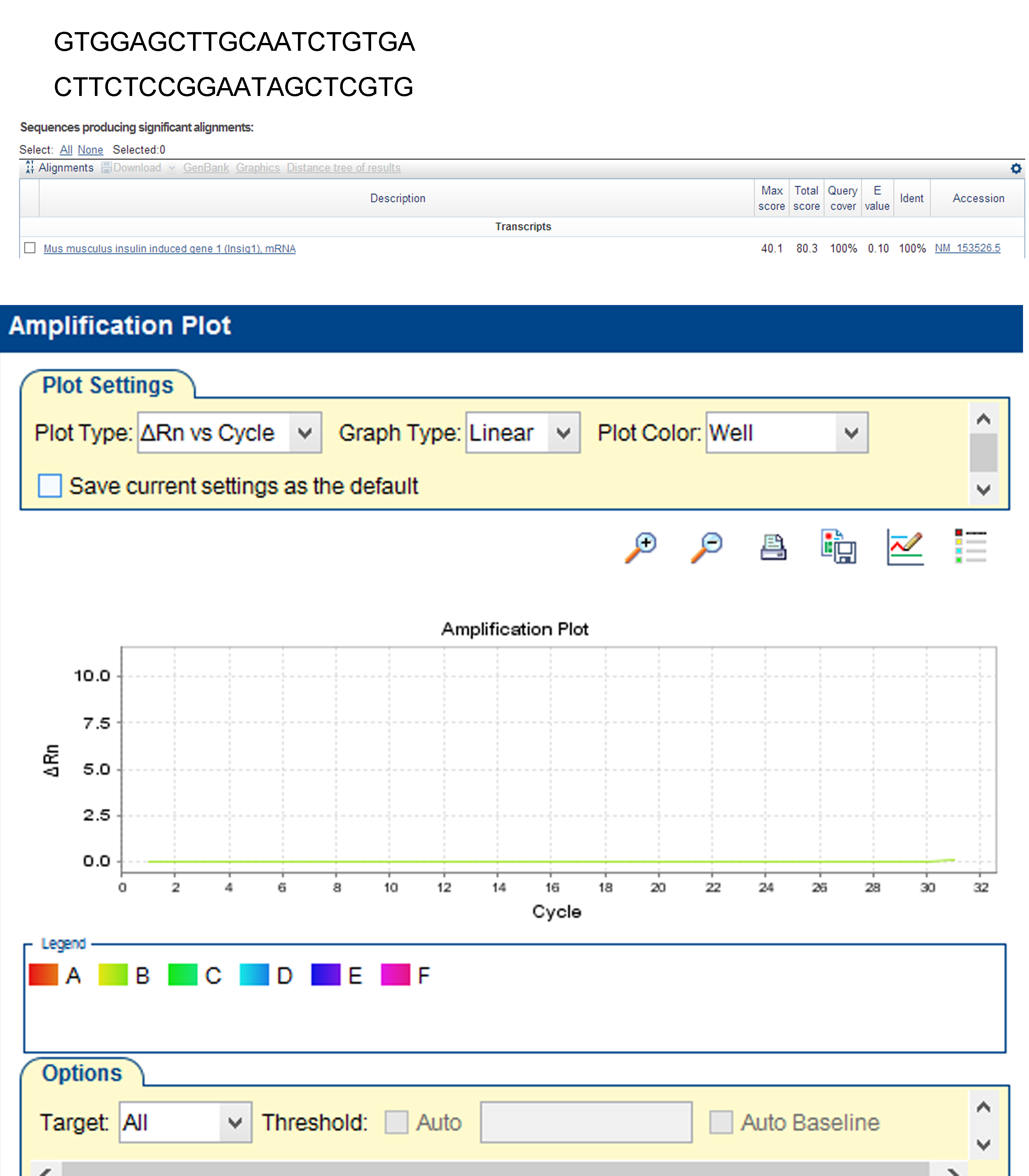

Supplement: S2 File — (ZIP) [file pone.0149670.s003.zip › S2 File/Insig1.tif]

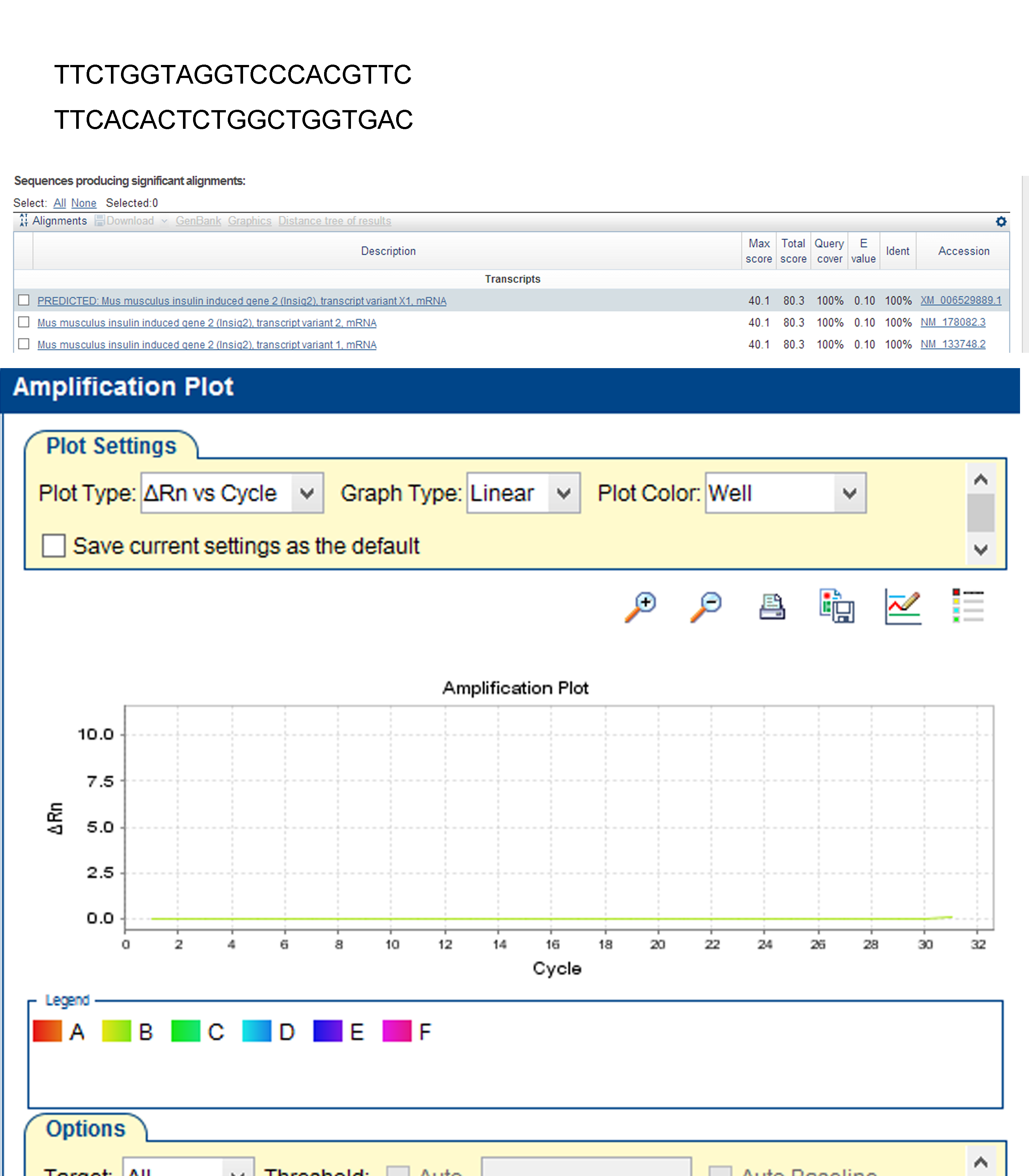

Supplement: S2 File — (ZIP) [file pone.0149670.s003.zip › S2 File/Insig2.tif]

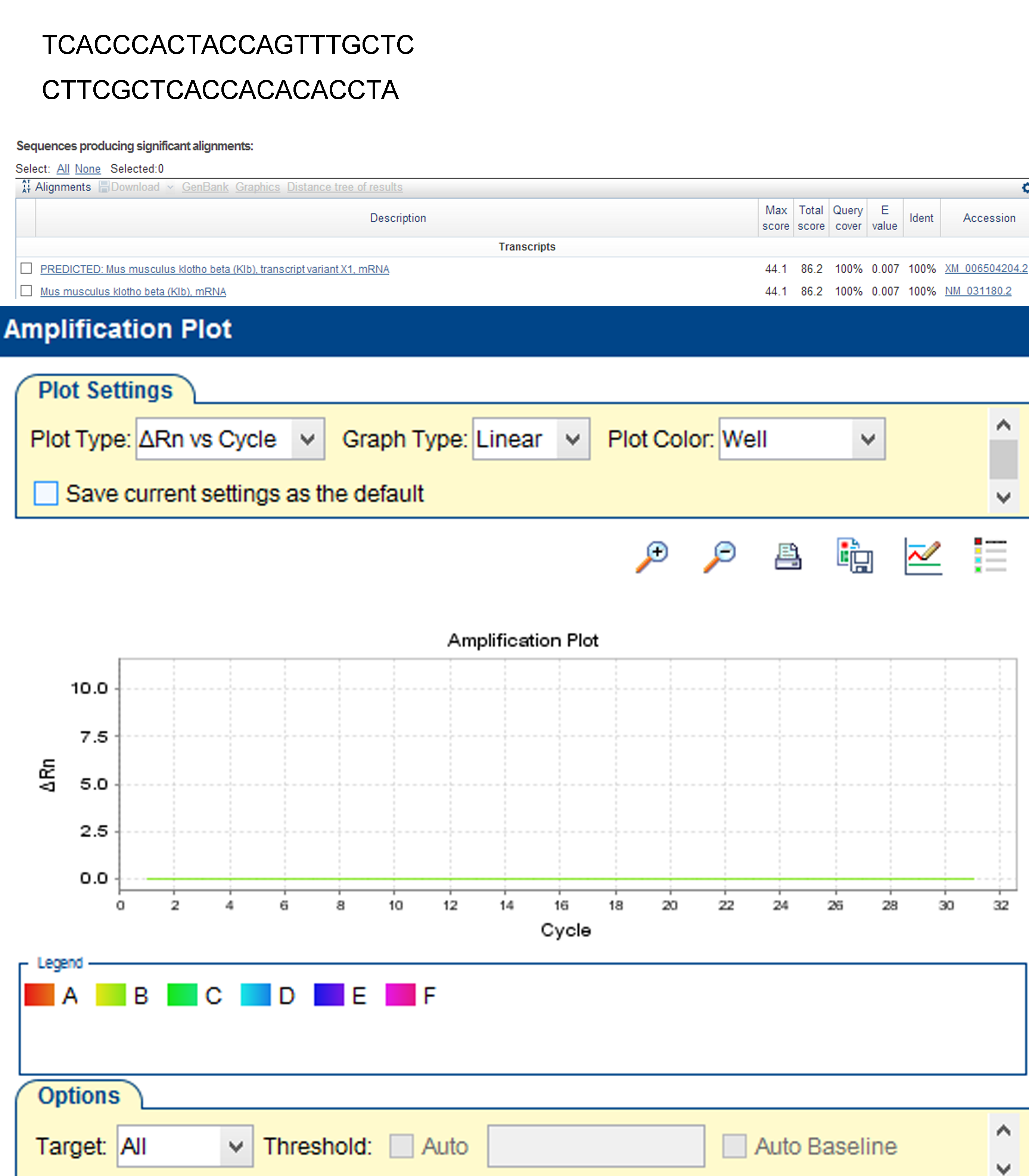

Supplement: S2 File — (ZIP) [file pone.0149670.s003.zip › S2 File/Klb.tif]

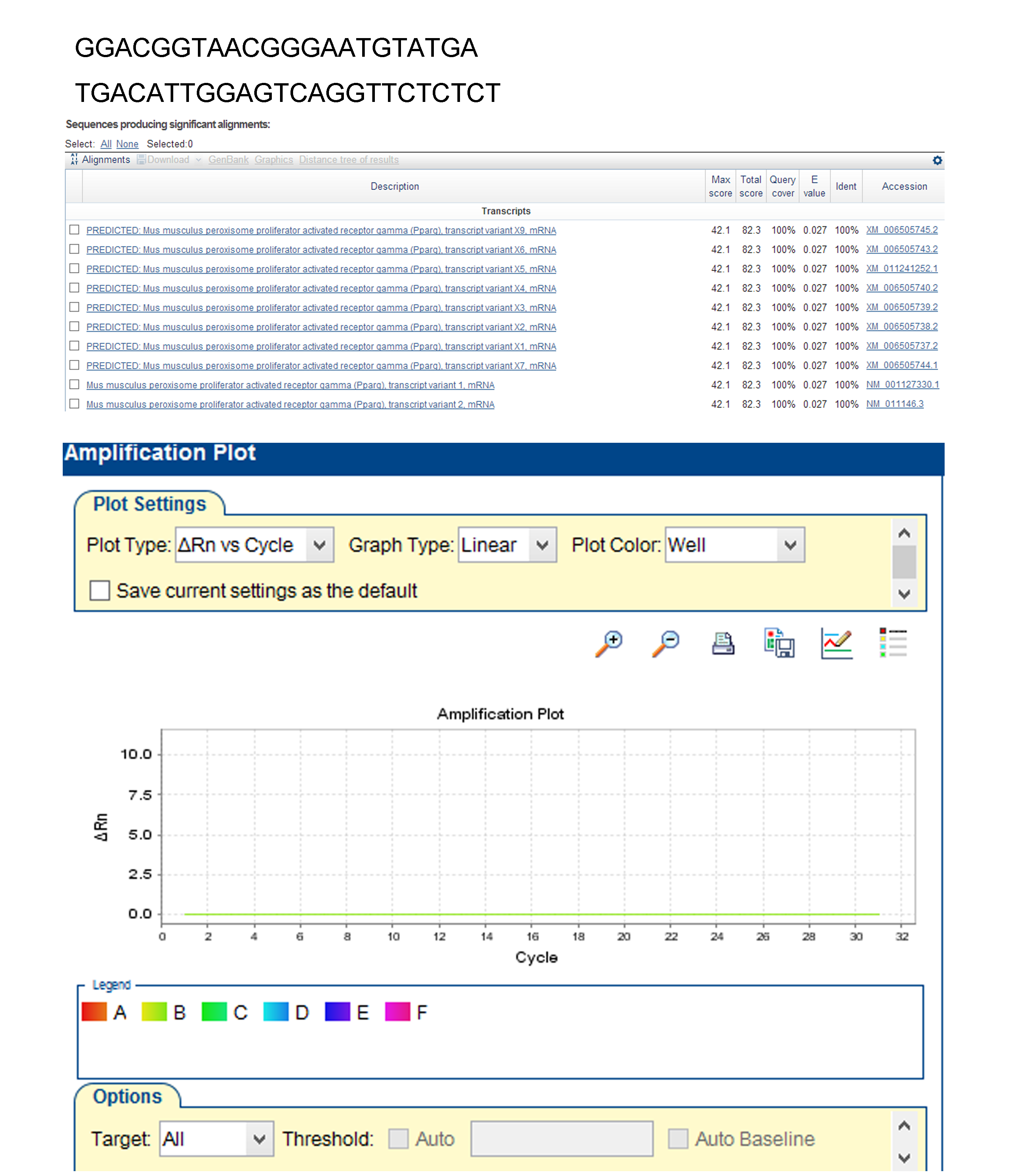

Supplement: S2 File — (ZIP) [file pone.0149670.s003.zip › S2 File/LPL.tif]
