# Supplementary material for: White Pitaya (Hylocereus undatus) Juice Attenuates Insulin Resistance and Hepatic Steatosis in Diet-Induced Obese Mice
Source: PLoS One. 2016 Feb 25;11(2):e0149670. doi: 10.1371/journal.pone.0149670 (PMC4767368; doi:10.1371/journal.pone.0149670)
Supplement: S1 Table — (DOCX) [file pone.0149670.s004.docx]

**S1 Table. Ingredients of purified diets (%)**

| Composition | LFD (10% Fat) | HFD (45% Fat) |
| --- | --- | --- |
| Sucrose | 33.17 | 20.14 |
| Corn starch | 29.86 | 8.48 |
| Lard | 1.9 | 20.68 |
| Casein | 18.96 | 23.31 |
| Maltodextrin | 3.32 | 11.65 |
| Cellulose | 4.74 | 5.83 |
| Soybean oil | 2.37 | 2.91 |
| Phosphate dicalcium | 1.23 | 1.51 |
| Potassium citrate | 1.56 | 1.92 |
| Vitamin Mix | 0.95 | 1.16 |
| L-cystein | 0.28 | 0.35 |
| Carbonate calcium | 0.5 | 0.64 |
| Mineral Mix | 0.95 | 1.16 |
| Choline bitartrate | 0.19 | 0.23 |
| Total | 99.98 | 99.97 |
| **Energy Composition** |  |  |
| Carbohydrate | 70 | 35 |
| Protein | 20 | 20 |
| Fat | 10 | 45 |
| Total | 100 | 100 |
