# Supplementary material for: White Pitaya (Hylocereus undatus) Juice Attenuates Insulin Resistance and Hepatic Steatosis in Diet-Induced Obese Mice
Source: PLoS One. 2016 Feb 25;11(2):e0149670. doi: 10.1371/journal.pone.0149670 (PMC4767368; doi:10.1371/journal.pone.0149670)
Supplement: S2 Table — (DOCX) [file pone.0149670.s005.docx]

**S2 Table. Primers used for real-time quantitative PCR**

| Gene | Sequence of forward primers (5' to 3') | Sequence of reverse primers (5' to 3') |
| --- | --- | --- |
| *Cpt1b* | TCGCAGGAGAAAACACCATGT | AACAGTGCTTGGCGGATGTG |
| *HL* | TGTGAATGTGGGGTTAGTGG | TCCAGCCATAGGAGAAGAGC |
| *LPL* | GGACGGTAACGGGAATGTATGA | TGACATTGGAGTCAGGTTCTCTCT |
| *Insig1* | GTGGAGCTTGCAATCTGTGA | CTTCTCCGGAATAGCTCGTG |
| *Insig2* | TTCTGGTAGGTCCCACGTTC | TTCACACTCTGGCTGGTGAC |
| *FGF21* | CGCAGTCCAGAAAGTCTCCT | ATTGTAACCGTCCTCCAGCA |
| *Klb* | TCACCCACTACCAGTTTGCTC | CTTCGCTCACCACACACCTA |
| *FGFR2* | AGCCACCAACCAAATACCAA | CCCCATCCTTAGTCCAACTG |
| *cFos* | GGATTTGACTGGAGGTCTGC | CGTTGCTGATGCTCTTGACT |
| *Egr1*  *HMGcoR* | ACCACCTTACCACCCACATC  GAGCGTGAACAAGGACCAAG | TATGCCTCTTGCGTTCATCA  CAGCCATTTTGCCAGAGTTT |
| *LDLR* | TGTGACCTTGTGGAACAGGA | AACGAAGCCATTTTCAGTGC |
| *Srebp1* | CACAGGTTCCCCATAGACAAA | GGAGGCAGAGAGCAGAGATG |
| *Pparγ* | CAGGAGCAGAGCAAAGAGGT | TGGACACCATACTTGAGCAGA |
| *Pparα* | CCTGCTTCCTGCCACTTG | GTTCACCCTGATTCCTGATGTC |
| *RPS18* | AGGATGTGAAGGATGGGAAG | TTCTTCAGCCTCTCCAGGTC |
| *ARBP* | ACCCTGAAGTGCTCGACATC | CCGATCTGCAGACACACACT |
| *TBP* | CCTTGTACCCTTCACCAATGAC | ACAGCCAAGATTCACGGTAGA |
| *B2M* | GGTCTTTCTGGTGCTTGTCTC | GTTCAGTATGTTCGGCTTCCC |
| *β-actin* | GTGCTATGTTGCTCTAGACTTCG | ATGCCACAGGATTCCATACC |
